# Characterization of LTR Retrotransposon Reverse Transcriptase in *Tamarix chinensis* L. and Activity Analysis Under Salt and Alkali Stresses

**DOI:** 10.3390/genes16111262

**Published:** 2025-10-26

**Authors:** Long Wang, Bo Li, Yuqian Wang, Shiji Wang, Meichun Zhang, Mengyao Li, Tong Zheng, Hongyan Wang

**Affiliations:** 1 Laboratory of Plant Epigenetics and Evolution, School of Life Sciences, Liaoning University, Shenyang 110036, China; 2 Academy of Agricultural and Forestry Sciences, Qinghai University, Xining 810016, China; 3 Key Laboratory of Molecular Epigenetics of the Ministry of Education, Northeast Normal University, Changchun 130024, China

**Keywords:** LTR-retrotransposon, reverse transcriptase (RT), abiotic stress, evolution, heterogeneity, *Tamarix chinensis* L.

## Abstract

Transposable elements (TEs) are major components of plant genomes and play crucial roles in adaptive genome evolution and stress tolerance. Under abiotic stress, activated TEs can generate abundant genetic variation and regulate the expression of stress-responsive genes. As a pioneer species in desert and saline–alkali environments, *Tamarix chinensis* L. has been little studied with respect to the abundance and evolutionary relationships of its LTR retrotransposons, particularly their activation patterns under salt and alkali stresses. This study aimed to investigate the characteristics of the reverse transcriptase (RT) domain of LTR retrotransposons in *T*. *chinensis* and to determine their patterns of activation in response to salt and alkali stresses. A total of 629 Ty1-*copia* and 607 Ty3-*gypsy* RT nucleotide sequences, which displayed high AT/GC ratios and evidence of stop codon insertions, were identified in *T*. *chinensis* by amplicon sequencing. Among these, 211 Ty1-*copia* and 117 Ty3-*gypsy* RT sequences with potential transpositional activity each contained distinct domains, suggesting a high degree of conservation. Phylogenetic analysis revealed that the RT sequences of *T*. *chinensis* are closely related to those of mangrove, wild potato, and *Ipomoea*, and may have undergone horizontal transfer. Expression analysis showed that 634 and 181 RT sequences were activated under salt and alkali stresses, respectively, with the majority belonging to salt-induced Ty1-*copia* families. Compared with the control group, under salt and alkali stresses, the cTy1-*copia* elements (Ty1-*copia* with amplificated from cDNA of *T*. *chinensis*, the same below) with dominant abundance were mainly concentrated in the Angela subfamily, while the cTy3-*gypsy* elements induced by alkali stress were primarily distributed in the Tekay and Reina subfamilies. Furthermore, four cTy1-*copia* and five cTy3-*gypsy* were identified as candidate key LTR retrotransposons responsive to salt and alkali stresses. Overall, this study provides new insights into the epigenetic mechanisms underlying the adaptation of *T*. *chinensis* to saline and alkali stresses and offers a theoretical basis for its potential applications in saline–alkali land reclamation.

## 1. Introduction

Transposable elements (TEs) are essential components of plant genomes and represent a major source of genetic variation [1,2,3]. Based on their mechanisms of action, TEs can be classified into DNA transposons and retrotransposons. Retrotransposons are further divided into long terminal repeat (LTR) and non-LTR retrotransposons, depending on the presence or absence of long terminal repeats. Among them, LTR retrotransposons are the most abundant in plant genomes, typically comprising two flanking LTRs and the *gag* and *pol* genes. The *pol* gene encodes key enzymes, including reverse transcriptase (RT), RNase H (RH), and integrase (INT) [4,5]. The conserved motifs within the RT domain serve as important markers for retrotransposon identification [6,7]. According to sequence similarity and the arrangement of retrotransposon-encoded enzymes, LTR retrotransposons can be further divided into the Ty1-*copia* and Ty3-*gypsy* superfamilies. In plant genomes, retrotransposons are first transcribed into RNA and then reverse-transcribed into cDNA under the catalysis of RT, after which the cDNA is inserted into new genomic locations. This unique mode of transposition has enabled their repeated amplification and selection over evolutionary time, resulting in typically high copy numbers [8]. For example, LTR retrotransposons account for approximately 60% of the tomato genome, 79.44% of maize, 54.52% of sorghum, and 25.78% of rice [9,10]. Previous studies have shown that frameshift mutations or the stop codon insertions in retrotransposons can cause loss of coding activity, explaining why most plant retrotransposons lack transpositional activity [11]. For instance, Rajput et al. cloned 36 Ty1-*copia* retrotransposons from pigeonpea, three of which contained one or more stop codons, ultimately resulting in loss of activity [12].

Retrotransposons play a vital role in plant responses to environmental stress [13,14]. They generally remain inactive under normal conditions; however, when exposed to stress, they can rapidly become activated and/or insert randomly into the genome, resulting in gene silencing or altered gene expression. Such insertions drive gene mutations, genome expansion, and ultimately species diversification [1,15,16]. For example, low-temperature activates Ty1-*copia* retrotransposons in blood orange, causing insertion near the *Ruby* locus, which enhances the expression of an *MYB* transcription factor, promotes anthocyanin biosynthesis, and results in the blood orange phenotype [17]. Similarly, insertion of the retrotransposon *Gret1* disrupts the *Vvmby1A* gene in grape, leading to the emergence of white grape cultivars. Subsequent rearrangement of the insertion site partially restored gene function, giving rise to red-skinned cultivars [18]. In *Arabidopsis*, the retrotransposon *ONSEN* is activated under heat stress and induces the upregulation of flanking genes, thereby mediating heat tolerance [19]. *GBRE-1*, a Ty1-*copia* retrotransposon, in the genome of *Gossypium barbadense*, which is activated under heat stress and results in the hairless stem phenotype [20]. He et al. reported that the retrotransposon *FaRE1* in cultivated strawberry (*Fragaria* × *ananassa*) can be activated by ABA, NAA, and 2,4-D, as well as by low-temperature stress, thereby contributing to cold-stress responses [21]. In addition, a Ty3-*gypsy* retrotransposon in *Cryptomeria japonica* is activated under heat stress, conferring enhanced stress tolerance [22]. Collectively, these findings suggest that retrotransposons activated by abiotic stress enhance plant tolerance to adverse conditions and play a crucial role in adaptive evolution and genetic diversity.

Environmental fluctuations profoundly affect plant growth and development, and plants must display phenotypic plasticity to adapt to such changes [23,24,25]. Retrotransposons activated under abiotic stress can further generate the genetic diversity required for evolutionary adaptation [14,26]. *Tamarix chinensis* L., a woody pioneer species of saline–alkali soils, plays important roles in soil improvement, windbreak and sand fixation, as well as stress tolerance and environmental remediation [27,28]. However, little is known about its molecular mechanisms of stress resistance, particularly the characteristics of retrotransposons activated under salt and alkali stresses. In this study, amplicon sequencing was employed to comprehensively identify the abundance and sequence features of LTR retrotransposon RT domains in *T. chinensis*, and to analyze their activity under salt and alkali stresses. These findings provide a foundation for understanding the transcriptional activity and functional roles of retrotransposons in *T. chinensis*, offering new insights into its unique tolerance mechanisms and potential applications in saline–alkali land reclamation.

## 2. Materials and Methods

### 2.1. Plant Materials and Salt/Alkali Stress Treatments

One-year-old branches of *T*. *chinensis* were collected, surface-sterilized, and cut into segments approximately 15 cm in length and 1 cm in diameter. The cuttings were planted in pots containing a substrate mixture of peat, vermiculite, and perlite (1:2:1, V:V:V). Plants were cultivated in a controlled walk-in growth chamber at 25 °C with 65–70% relative humidity under a 15 h light/9 h dark photoperiod. After 50 days of cultivation, uniformly developed cuttings were selected for stress treatments. Control and treatment groups were established, each with five biological replicates. For stress induction, neutral salts (Na_2_SO_4_ and NaCl) and alkali salts (Na_2_CO_3_ and NaHCO_3_) were mixed at a 1:1 molar ratio to prepare salt and alkali solutions, respectively. The final concentrations were 200 mM for the salt treatment and 100 mM for the alkali treatment. After 10 days of stress exposure, leaf tissues were harvested, immediately frozen in liquid nitrogen, and stored at –80 °C for subsequent analyses.

### 2.2. Amplification and Sequencing of LTR Retrotransposons in T. chinensis

Genomic DNA was extracted from *T. chinensis* using a modified CTAB method [29]. Total RNA was isolated and purified from control, salt-treated, and alkali-treated samples using the Plant RNA Kit (R6827-00, OMEGA Bio-Tek, Norcross, GA, USA). First-strand cDNA was synthesized from RNA using the PrimeScriptTM RT reagent kit (TaKaRa, Shiga, Japan). Both genomic DNA and cDNA were used as templates for amplification with degenerate primers specific to Ty1-*copia* (Forward: 5′-CARATGGAYGTNAARAC-3′; Reverse: 5′-CATRTCRTCNACRTA-3′) and Ty3-*gypsy* (Forward: 5′-MRNATGTGYGTNGAYTAYMG-3′; Reverse: 5′-RCAYTTNSWNARYTTNGCR-3′) retrotransposons [30,31]. The amplification products were used for library construction and subjected to paired-end (PE) sequencing on the Illumina MiSeq platform (Personalbio, Shanghai, China). Data quality was evaluated using FastQC (v0.11.7), including assessment of GC content distribution and average sequencing quality. Adapter contamination was removed using Adapter Removal (v2.0), and low-quality reads (Q < 15) or short reads (<50 bp) were filtered out using fastp (v0.20.0). After quality control, paired-end reads were merged using FLASH (v1.2.11). Three independent experiments were conducted.

### 2.3. Classification and Activity Screening of T. chinensis LTR Retrotransposons

Clustering analysis was performed using CD-Hit software (v4.8.1) with a similarity threshold of 93% [32]. Filtered nucleotide sequences were translated into amino acid sequences with DNAMAN (v9.0), followed by multiple sequence alignment and conserved domain analysis. Sequences containing frameshift mutations or stop codon insertions were excluded, and the remaining sequences were designated as retrotransposons with potential transcriptional activity for subsequent analyses.

### 2.4. Sequence Characterization of Ty1-copia and Ty3-gypsy RT Domains in T. chinensis

The AT/GC ratio of nucleotide sequences was calculated using the EditSeq function in DNASTAR (v11.1). Sequence length and copy number were determined with Python3 and Perl scripts (v3.13.0) (Table S1). Figures were generated and refined using Jalview (v2.11.4.0).

### 2.5. Phylogenetic Analysis of Ty1-copia and Ty3-gypsy in T. chinensis

Ty1-*copia* and Ty3-*gypsy* RT sequences used for TE classification were downloaded from the NCBI database (https://blast.ncbi.nlm.nih.gov/Blast.cgi, accessed on 25 March 2025) and Phytozome (https://phytozome.jgi.doe.gov/pz/portal.html, accessed on 25 March 2025), including those from *Populus trichocarpa*, *Pinus pinaster*, *Populus ciliata*, *Pinus cembra*, rice (*Oryza sativa*), *Arabidopsis thaliana*, papaya (*Carica papaya*), and tea (*Camellia sinensis*)**.** Papaya and tea belong to the same order (Ericales) as *T. chinensis* (Tables S2 and S3) [33,34]. Phylogenetic trees were constructed from amino acid sequences using the neighbor-joining (NJ) method in MEGA (v11), with 1000 bootstrap replicates; all other parameters were left at their default settings.

### 2.6. Analysis of Ty1-copia and Ty3-gypsy Dominant Abundance Under Salt and Alkali Stresses

*cTy1-copia* and *cTy3-gypsy* elements derived from amplicon sequencing of cDNA from control, salt-stressed, and alkali-stressed *T*. *chinensis* samples were used to analyze expression patterns. cDNA libraries were constructed from *T*. *chinensis* samples under control, salt stress, and alkali stress conditions, followed by paired-end amplicon sequencing on the Illumina NovaSeq platform. Data quality was assessed using FastQC (v0.11.7), including evaluation of GC content distribution and average sequencing quality. Adapter contamination was removed using Adapter Removal (v2.0), and low-quality reads (Q < 15) or short reads (<50 bp) were filtered out using fastp (v0.20.0). After quality control, paired-end reads were merged using FLASH (v1.2.11). Finally, VSEARCH (v2.15.1) was employed to perform statistical comparisons of read abundance among different treatment groups. Three independent experiments were conducted.

## 3. Results

### 3.1. Clustering and Characterization of LTR Retrotransposon RT Domains in T. chinensis

**Figure 1:**
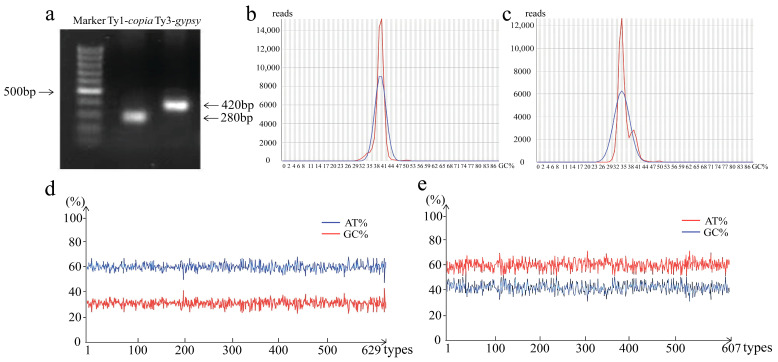
Sequence characteristics of LTR retrotransposon RT domains in the *T. chinensis* genome. (**a**) Amplification profiles of Ty1-*copia* and Ty3-*gypsy* retrotransposons; (**b**) GC content distribution of Ty1-*copia*; (**c**) GC content distribution of Ty3-*gypsy*. Note: the red line represents the observed distribution curve, and the blue line represents the theoretical distribution curve; (**d**) AT and GC base composition of Ty1-*copia* families (TC*copia1*-TC*copia629*); (**e**) AT and GC base composition of Ty3-*gypsy* families (TC*gypsy1*-TC*gypsy607*).

Retrotransposon domains typically contain multiple conserved motifs that are essential for the catalytic activity of RT, making these conserved sequences important markers for retrotransposon identification [6,7]. Using degenerate primers previously developed for plant LTR retrotransposons, RT conserve domains of Ty1-*copia* and Ty3-*gypsy* were successfully amplified from the *T. chinensis* genome. As shown in Figure 1a, the amplified fragments were approximately 280 bp (Ty1-*copia*) and 420 bp (Ty3-*gypsy*), consistent with earlier reports, confirming that these primer sets are suitable for amplifying RT sequences of *T. chinensis* Ty1-*copia* and Ty3-*gypsy* retrotransposons [30,31]. Amplicon sequencing yielded 251,930 reads for Ty1-*copia* and Ty3-*gypsy* combined, with Q30 values exceeding 90% (Figure 1b,c). After quality control and sequence assembly, 84,747 Ty1-*copia* and 47,011 Ty3-*gypsy* sequences were obtained.

Given the high sequence similarity and copy number of LTR retrotransposons in plant genomes [8], subfamily clustering was performed. A total of 629 distinct Ty1-*copia* families (TC*copia1*-TC*copia629*) and 607 distinct Ty3-*gypsy* families (TC*gypsy1*-TC*gypsy607*) were identified. Previous studies have suggested that a high AT/GC ratio in LTR retrotransposon promotes transpositional activity [35]. In this study, 99.7% of Ty1-*copia* and 99.5% of Ty3-*gypsy* exhibited AT/GC ratios greater than 1, indicating that most LTR retrotransposons in *T. chinensis* may possess potential transpositional activity (Figure 1d,e).

### 3.2. Activity and Conserved Domain Analysis of LTR Retrotransposons

**Figure 2:**
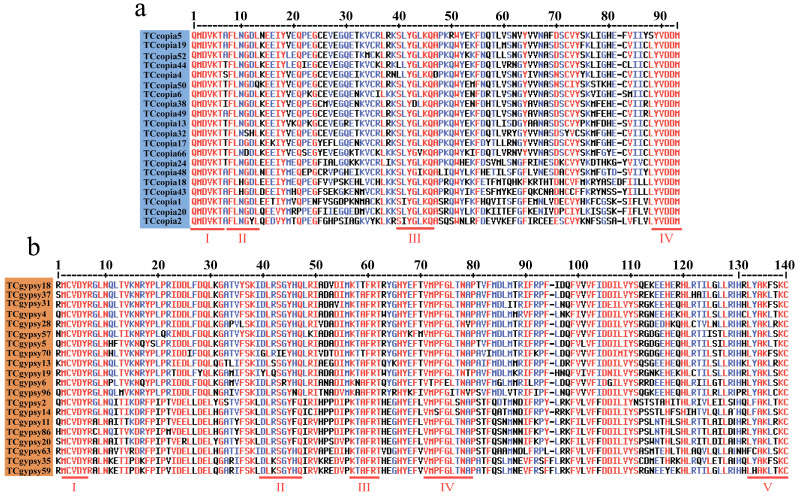
Conserved domain analysis of LTR retrotransposon RT sequences in *T. chinensis* (**a**) Conserved domain analysis of Ty1-*copia* (representative sequences shown); (**b**) conserved domain analysis of Ty3-*gypsy* (representative sequences shown).

Frameshift mutations or the insertion of stop codons in the RT domain of LTR retrotransposons can result in loss of transpositional activity [12,36]. Sequence alignment showed that among Ty1-*copia* retrotransposons, 418 sequences (66.45%) contained stop codons, while 211 sequences (33.55%) did not. For Ty3-*gypsy* retrotransposons, 492 sequences (80.79%) contained stop codons, and 117 sequences (19.21%) did not. Notably, the RT sequences of Ty1-*copia* and Ty3-*gypsy* retrotransposons without stop codons contained highly conserved domains (Figure 2). Specifically, the 211 Ty1-*copia* RT sequences contained four conserved domains: QMDVKT (I) at the 5′ end, YVDDM (IV) at the 3′ end, and FLNGDL (II) and SLYGLKQ (III) in the middle. Among these, QMDVKT and YVDDM were the most highly conserved, while FLNGDL and SLYGLKQ exhibited partial amino acid substitutions (Figure 2a). The 117 Ty3-*gypsy* RT sequences contained five conserved domains: MCVDY (I) at the 5′ end, LYAKLXKC (V) at the 3′ end, and three newly identified conserved domains in between, DLRSGYHQ (II), KTAFRT (III), and VMPFGLTNAP (IV). Except for the 5′ MCVDY domain, all other conserved domains displayed partial amino acid substitutions (Figure 2b).

### 3.3. Phylogenetic Analysis of LTR Retrotransposons Across Species

**Figure 3:**
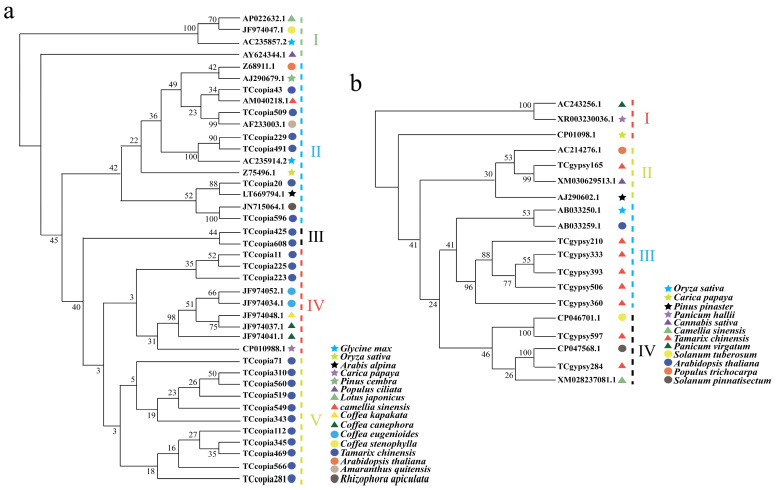
Phylogenetic analysis of LTR retrotransposon RT sequences across species in *T. chinensis*. (**a**) Phylogenetic analysis of Ty1-*copia* retrotransposons; (**b**) phylogenetic analysis of Ty3-*gypsy* retrotransposons. Phylogenetic trees were constructed using the NJ method with 1000 bootstrap replicates, and all other parameters set to default.

To further clarify the evolutionary relationships of Ty1-*copia* and Ty3-*gypsy* retrotransposons in *T. chinensis*, phylogenetic analyses were performed using RT sequences from *T. chinensis* and representative species (Figure 3a). Ty1-*copia* retrotransposons were divided into five clades, with TC*copia43*, TC*copia509*, TC*copia596*, and TC*copia20* from *T. chinensis* clustering with tea, *A*. *quitensis*, mangrove (*Rhizophora*), and *A*. *alpina*, respectively, suggesting close evolutionary relationships between them. Similarly, Ty3-*gypsy* retrotransposons from *T. chinensis*, together with those from *P*. *trichocarpa*, *P*. *pinaster*, *A*. *thaliana*, *O*. *sativa*, *C*. *papaya*, and *C*. *sinensis*, were divided into four clades. Among them, TC*gypsy165* clustered with *C*. *sativa*, while TC*gypsy210*, TC*gypsy333*, TC*gypsy506*, TC*gypsy393*, TC*gypsy360*, TC*gypsy284*, and TC*gypsy597* clustered with wild potato (*Solanum pinnatisectum*), *Ipomoea trifida*, *Arabidopsis*, and rice (Figure 3b). In addition, TC*copia596* shared 91.27% sequence similarity with retrotransposon JN715064.1 from *Rhizophora apiculata* (same class but different order), and both clustered in the same clade. TC*gypsy165* showed 90.10% similarity with *C*. *sativa* XM_030629513.1, TC*gypsy284* showed 91.35% similarity with *S*. *pinnatisectum* CP047568.1, and TC*gypsy597* showed 92.07% similarity with *S*. *tuberosum* P046701.1, all clustering in the same evolutionary branches. These findings suggest that LTR retrotransposons in *T. chinensis* exhibited high similarity to those in other distantly related species, and some of them may have undergone horizontal transfer with the above species [35].

### 3.4. Sequencing and Clustering Analysis of LTR Retrotransposons in T. chinensis Under Salt and Alkali Stresses

**Figure 4:**
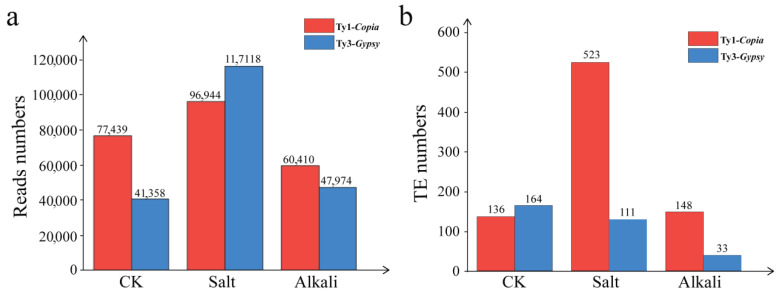
Abundance of transcriptionally active LTR retrotransposons in the genome of *T. chinensis* under salt and alkali stresses. (**a**) Read counts of cTy1-*copia* and cTy3-*gypsy* retrotransposons after quality control and paired-end sequence assembly; (**b**) numbers of cTy1-*copia* and cTy3-*gypsy* retrotransposon subfamilies with transcriptional activity. Note: CK denotes the untreated control group.

TEs with transcriptional or transpositional activity play crucial roles in adaptive genome evolution and abiotic stress responses in plants [37,38]. As a pioneer species tolerant to salt and alkali stresses [27,28], *T. chinensis* provides an important system for investigating the transcriptional characteristics of LTR retrotransposons and their potential roles in stress resistance mechanisms. Based on preliminary observations that *T. chinensis* exhibited the strongest response at 200 mM salt stress and 100 mM alkali stress (tolerance threshold, unpublished data), amplicon sequencing was performed on cDNA-derived Ty1-*copia* and Ty3-*gypsy* retrotransposons under these conditions. The numbers of cTy1-*copia* reads in the control, salt-stressed, and alkali-stressed samples were 77,439, 96,944, and 60,410, respectively, while the corresponding numbers of cTy3-*gypsy* reads were 41,358, 117,118, and 47,974 (Figure 4a).

Following clustering, de-redundancy, and conserved domain analysis, the numbers of active cTy1-*copia* elements in the control, salt-stressed, and alkali-stressed samples were 136, 523, and 148, respectively, whereas the numbers of active cTy3-*gypsy* elements were 164, 111, and 33 (Figure 4b). Notably, transcriptionally active retrotransposons were far more abundant under salt stress than under alkali stress, indicating a stronger transcriptional response to salt. Furthermore, across both stress conditions, the total number of active cTy1-*copia* elements (671) was 4.66 times greater than that of cTy3-*gypsy* elements (144), highlighting a marked specificity. These findings suggest that Ty1-*copia* retrotransposons may possess greater adaptability and responsiveness under adverse conditions, implying their more prominent role in the stress response of *T. chinensis*.

### 3.5. Expression Specificity of LTR Retrotransposons in T. chinensis Under Salt and Alkali Stresses

**Figure 5:**
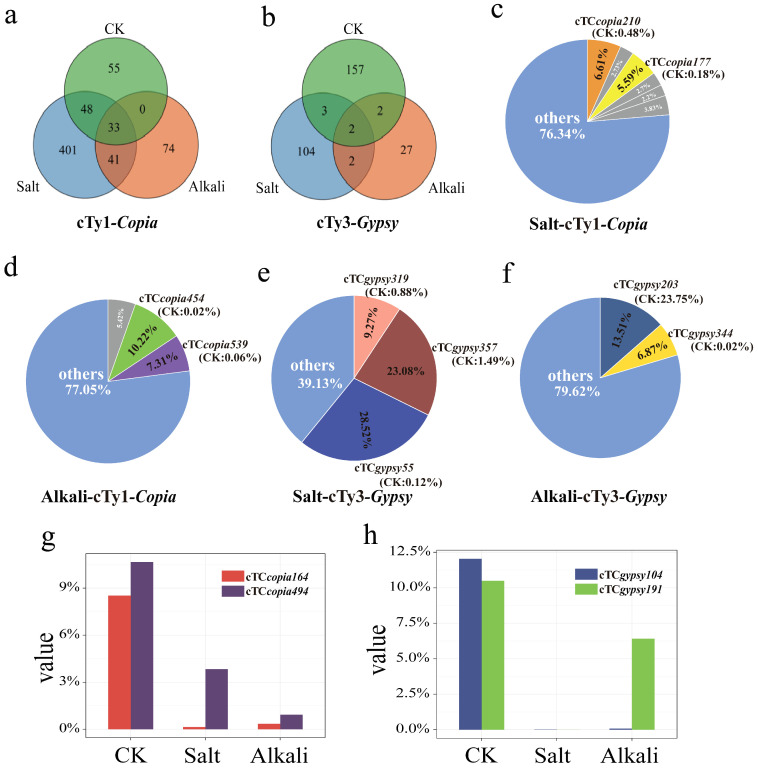
Analysis of dominant cTy1-*copia* and cTy3-*gypsy* retrotransposons in *T*. *chinensis*. (**a**) Schematic diagram of cTy1-*copia* elements activated under salt and alkali stresses; (**b**) schematic diagram of cTy3-*gypsy* elements activated under salt and alkali stresses; (**c**) dominant cTy1-*copia* elements under salt stress; (**d**) dominant cTy1-*copia* elements under alkali stress; (**e**) dominant cTy3-*gypsy* elements under salt stress; (**f**) dominant cTy3-*gypsy* elements under alkali stress; (**g**) response patterns of cTy1-*copia* under different treatments; (**h**) response patterns of cTy3-*gypsy* under different treatments. Note: CK denotes the untreated control group.

To elucidate the specific response patterns of retrotransposons activated under salt and alkali stresses, the transcriptionally active LTR retrotransposon RT sequences identified above were further analyzed. As shown in Figure 5a, 634 retrotransposons (523 + 111) were activated under salt stress, while 181 retrotransposons (148 + 33) were activated under alkali stress. Among them, 401 cTy1-*copia* retrotransposons were specifically expressed under salt stress, 74 were specifically expressed under alkali stress, and 41 responded to both stresses. Similarly, 104 cTy3-*gypsy* retrotransposons were specifically expressed under salt stress, 27 under alkali stress, and only 2 responded to both (Figure 5b). Through analysis of dominant transposable elements (TEs), it was found that under salt stress, two highly abundant cTy1*-copia* elements (cTC*copia210* and cTC*copia177*) were detected, both of which showed low abundance in the control group (<0.5%) (Figure 5c). Similarly, under alkali stress, two dominant cTy1-*copia* elements (cTC*copia454* and cTC*copia539*) were identified, accounting for only 0.02% and 0.06% in the control group, respectively (Figure 5d). Compared with cTy1*-copia*, cTy3*-gypsy* appeared to respond more strongly to salt and alkali stresses. For instance, three dominant cTy3-*gypsy* elements (cTC*gypsy319*, cTC*gypsy357*, and cTC*gypsy55*) were present under salt stress. Notably, cTC*gypsy357* and cTC*gypsy55* exhibited high abundance (>23%) under salt stress but accounted for only 1.49% and 0.12% in the control group, respectively (Figure 5e). Under alkali stress, two dominant cTy3-*gypsy* elements (cTC*gypsy203* and cTC*gypsy344*) were identified; interestingly, cTC*gypsy203* already had a relatively high proportion in the control group (23.75%), whereas cTC*gypsy344* accounted for only 0.02% (Figure 5f).

In addition, cTC*copia164* and cTC*copia494* showed dominant abundance in the control group but declined under stress treatments, particularly under alkali stress (Figure 5g). Similarly, cTC*gypsy104* and cTC*gypsy191* were dominant in the control group but exhibited low abundance in the treatment groups; unlike the alkali stress treatment, both were nearly undetectable under salt stress (Figure 5h). Furthermore, sequence feature analysis of dominant LTR retrotransposons under salt and alkali stresses revealed that their AT/GC ratios were all greater than 1 (ranging from 1.01 to 1.72). Collectively, these results indicate that LTR retrotransposons show differential responses to distinct abiotic stresses in *T*. *chinensis*, and that cTy3-*gypsy* elements may be more active under salt stress compared with alkali stress.

### 3.6. Phylogenetic Analysis of LTR Retrotransposons in T. chinensis Under Salt and Alkali Stresses

**Figure 6:**
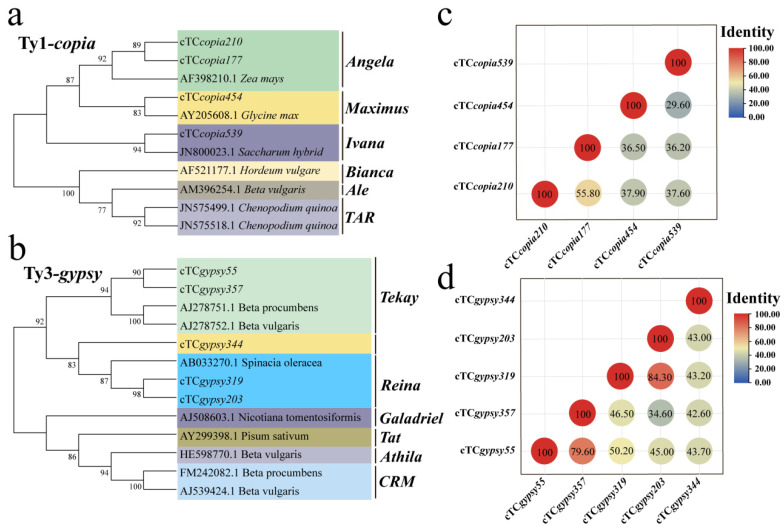
Phylogenetic analysis of dominant LTR retrotransposons in *T. chinensis* under salt and alkali stresses. (**a**) Phylogenetic relationships of cTy1-*copia* retrotransposons responsive to salt and alkali stresses; (**b**) phylogenetic relationships of cTy3-*gypsy* retrotransposons responsive to salt and alkali stresses. Phylogenetic trees were constructed using the NJ method with 1000 bootstrap replicates; all other parameters were set to default. (**c**) Sequence similarity analysis of cTy1-*copia* retrotransposons responsive to salt and alkali stresses; (**d**) sequence similarity analysis of cTy3-*gypsy* retrotransposons responsive to salt and alkali stresses.

Previous studies have shown that Ty1-*copia* retrotransposons comprise six subfamilies: Tork/TAR, Retrofit/Ale, Tork/Angela, Oryco/Ivana, Bianca, and Sire/Maximus [39]. Similarly, Ty3-*gypsy* retrotransposons include six subfamilies: Del/Tekay, Reina, Galadriel, CRM, Athila, and Tat [40]. To investigate the subfamily classification of dominant LTR retrotransposons under salt and alkali stresses, previously reported Ty1-*copia* and Ty3-*gypsy* RT domain amino acid sequences were retrieved from the GenBank database as reference sequences. Phylogenetic analysis was performed by these reference sequences with the amino acid sequences of the dominant cTy1-*copia* (4) and cTy3-*gypsy* (5) retrotransposons in *T*. *chinensis*. The results revealed that salt-responsive cTy1-*copia* sequences clustered mainly within the Angela subfamily, whereas alkali-responsive cTy1-*copia* sequences were primarily grouped within the Maximus and Ivana subfamilies (Figure 6a). Similarly, salt-responsive cTy3-*gypsy* sequences were clustered into the Tekay and Reina subfamilies, while the alkali-responsive cTy3-*gypsy* element (cTC*gypsy203*) was also grouped into the Reina subfamily (Figure 6b).

In addition, stress-activated LTR retrotransposons exhibited notable sequence similarities. For example, cTC*gypsy319* (salt-responsive) and cTC*gypsy203* (alkali-responsive) shared 84.30% similarity (Figure 6d), indicating that they originated from the same retrotransposon families. Together, these findings suggest that Ty1-*copia* and Ty3-*gypsy* retrotransposons in *T. chinensis* exhibit both commonalities and differences in their responses to salt and alkali stresses. Among the subfamilies, Angela, Tekay, and Reina appear to play particularly important roles in regulating stress adaptation.

## 4. Discussion

### 4.1. Sequence Characteristics of LTR Retrotransposons in the T. chinensis Genome

TEs are important components of plant genomes and play critical roles in plant responses to environmental stress [13,14,41]. As a pioneer species in deserts and saline–alkali soils, research on *T. chinensis* has largely focused on rhizosphere microbial interactions and functional gene discovery [28,42]. However, epigenetic studies of *T. chinensis*, such as the identification of retrotransposons in its genome and their activation under abiotic stress, remain scarce.

Given the high copy numbers and extensive polymorphism of LTR retrotransposons, their conserved RT domains were amplified and sequenced. In *T. chinensis*, the nucleotide lengths of Ty1-*copia* RT sequences ranged from 261 to 291 bp, while those of Ty3-*gypsy* RT sequences ranged from 390 to 474 bp (Figure 1a). Sequence similarity analysis showed that Ty1-*copia* RT sequences shared 21.01–92.83% similarity, whereas Ty3-*gypsy* RT sequences shared 17.98–92.80% similarity. Similarly, Zhou et al. cloned 165 Ty3-*gypsy* RT sequences from four bamboo species, with lengths ranging from 366 to 438 bp and similarities of 52.2–99.8% [43]. These findings suggest that polymorphisms in sequence length and nucleotide composition are likely important contributors to the heterogeneity of plant LTR retrotransposons.

### 4.2. Transpositional Activity and Phylogenetic Relationships of Ty1-copia and Ty3-gypsy in T. chinensis

Previous studies have shown that the RT domains of plant retrotransposons are typically enriched in AT bases relative to GC bases, and higher AT content can enhance DNA flexibility [35]. In *T. chinensis*, analysis of AT/GC ratios revealed that 99.7% of Ty1-*copia* sequences and 99.5% of Ty3-*gypsy* sequences had ratios greater than 1 (Figure 1d,e), suggesting potential transpositional activity. During genome evolution, frequent frameshift mutations and the insertion of stop codons can lead to loss of retrotransposon activity [12,36]. Sequence comparisons indicated that 66.45% of Ty1-*copia* and 33.55% of Ty3-*gypsy* retrotransposon sequences in *T. chinensis* contained stop codon insertions. Such frameshift mutations and premature terminations may cause loss of coding capacity, potentially serving as a mechanism to mitigate harmful effects of retrotransposons on plant genomes [44]. Horizontal transfer of transposons, which involves movement across species boundaries, is considered an important driver of plant genome evolution. Moaine El Baidouri et al. proposed similarity thresholds for identifying interspecies horizontal transfer events: 85% for inter-class and 90% for inter-order comparisons [44].

In *T. chinensis*, we detected RT sequences with up to 90% similarity to those of distantly related species (same class, different orders), such as TC*copia596* with *Rhizophora*, TC*gypsy284* with wild potato (*S*. *pinnatisectum*), and TC*gypsy597* with *I*. *trifida*, all of which clustered within the same phylogenetic branches (Figure 3a, b). These results provide candidate sequences supporting potential horizontal transfer events of retrotransposons between *T. chinensis* and other species.

### 4.3. LTR Retrotransposon Activity and Dominance in T. chinensis Under Salt and Alkali Stresses

Abiotic stresses can activate transposons, shifting them from a silenced to an active state and thereby influencing not only their own expression but also the regulation of stress-responsive genes [1,13,14]. In *T. chinensis*, the number of active LTR retrotransposon sequences under salt stress (634) was markedly higher than under alkali stress (181), suggesting distinct response mechanisms and highlighting the potential role of active LTR retrotransposons in salt tolerance. Notably, compared with the 144 activated cTy3-*gypsy* retrotransposons, a larger number of active cTy1-*copia* retrotransposons (671) were detected across both stress conditions (Figure 4b), implying that Ty1-*copia* elements may be more responsive to abiotic stress [38]. Although our analysis was based on a simple abundance comparison of sequencing reads, clear dominant sequences were nevertheless observed in the treatment groups compared with the control. Notably, the number of dominant cTy1-*copia* and cTy3-*gypsy* elements under salt stress was higher than that under alkali stress. These findings highlight distinct regulatory strategies adopted by plants in response to different abiotic stresses and provide evidence that LTR retrotransposons contribute to the adaptive evolution of salt tolerance in *T*. *chinensis*. Further analysis of upregulated sequences under both stresses revealed consistently high AT/GC ratios (1.01–1.72) (Figure 5). In particular, salt-responsive LTR retrotransposons appeared to be more active, especially cTy3-*gypsy* elements such as cTC*gypsy357* and cTC*gypsy55*, suggesting that Ty3-gypsy may play a potentially important role in the stress adaptation of *T*. *chinensis* under salt stress.

### 4.4. Characterization Analysis of LTR Retrotransposons Under Salt and Alkali Stresses

To further investigate similarities and differences in the response patterns of LTR retrotransposons under salt and alkali stresses, phylogenetic analyses were performed on the dominant sequences identified under stress conditions. The results showed that salt-responsive cTy1*-copia* sequences were clustered into the Angela subfamily, whereas alkali-responsive cTy1-*copia* sequences were grouped into the Maximus and Ivana subfamilies (Figure 6a). Similarly, salt-responsive cTy3-*gypsy* sequences were clustered into the Tekay and Reina subfamilies, while alkali-responsive cTy3-*gypsy* sequences were grouped into the Reina subfamily (Figure 6b), indicating that *T*. *chinensis* exhibits differential responses to salt and alkali stresses. Sequence similarity analysis further revealed that certain elements shared high similarity (>80%) between salt- and alkali-responsive groups, such as *cTCgypsy319* (salt-responsive) and *cTCgypsy203* (alkali-responsive). These findings suggest that retrotransposons may possess multiple response capacities under different stress conditions, providing new insights into the functional diversity of transposable elements in plant stress adaptation.

## 5. Conclusions

Using degenerate primers and amplicon sequencing, this study examined the sequence characteristics, evolutionary relationships, and stress-responsive patterns of LTR retrotransposon RT domains in *T. chinensis*. A total of 629 Ty1-*copia* and 607 Ty3-*gypsy* RT nucleotide sequences were identified, many of which exhibited high AT/GC ratios and stop codon insertions. Phylogenetic analysis indicated that *T. chinensis* LTR retrotransposon RT sequences may have undergone horizontal transfer. Under salt and alkali stresses, retrotransposon activation was more pronounced under salt conditions, with some RT sequences responding to both stresses. Dominant cTy1-*copia* elements were mainly clustered within the Angela subfamily, while cTy3-*gypsy* elements were predominantly grouped into the Tekay and Reina subfamilies. Notably, four cTy1-*copia* and five cTy3-*gypsy* were identified as candidate key LTR retrotransposons responsive to salt and alkali stresses. These findings provide preliminary theoretical evidence for the contribution of retrotransposons to stress tolerance in *T. chinensis* and offer new insights into the potential mechanisms underlying its adaptive responses to adverse environments.

## Data Availability

The original contributions presented in this study are included in the article/Supplementary Materials. Further inquiries can be directed to the corresponding author.

## Supplementary Materials

The following supporting information can be downloaded at https://www.mdpi.com/article/10.3390/genes16111262/s1, Table S1. Perl scripts. Table S2. Comparison Results of Ty1-*copia* Transposon in NCBI. Table S3. Comparison Results of Ty3*-gypsy* Transposon in NCBI.
