# Supplementary material for: Characterization of LTR Retrotransposon Reverse Transcriptase in Tamarix chinensis L. and Activity Analysis Under Salt and Alkali Stresses
Source: Genes (Basel). 2025 Oct 26;16(11):1262. doi: 10.3390/genes16111262 (PMC12651991; doi:10.3390/genes16111262)
Supplement: Supplementary file 1 [file genes-16-01262-s001.zip › Table S1.pdf]

Table S1. Perl scripts.

```
#!/bin/python3

import os
import re
import sys
import glob

data_path = './'
seq_length = 100
'''
for file in glob.glob(f'{data_path}/*fa'):
	out_file = open(file+'.filter.fasta', 'w')
	file_info = open(file, 'r')
	for line in file_info:
		if line.startswith('>'):
			if seq == '0':
				pass
			else:
				if len(seq) >= seq_length:
					out_file.write(f'{seq_id}\n{seq}\n')
			seq = ''
			seq_id = line.strip()
		else:
			seq += line.strip()
	if len(seq) >= seq_length:
		out_file.write(f'{seq_id}\n{seq}\n')
	file_info.close()
	out_file.close()
'''
seq_dict = {}
for file in glob.glob(f'{data_path}/*fa'):
	out_file = open(file+'.filter.fasta', 'w')
	file_info = open(file, 'r')
	for line in file_info:
		if line.startswith('>'):
			seq_index = '0'
			size_value = int(re.search('size=(\d{1,})', line.strip()).group(1))
			if size_value >= seq_length:
				seq_index = '1'
				out_file.write(line)
		else:
			if seq_index == '1':
```

```
        out_file.write(line)
file_info.close()
out_file.close()
```
