# Supplementary material for: Characterization of LTR Retrotransposon Reverse Transcriptase in Tamarix chinensis L. and Activity Analysis Under Salt and Alkali Stresses
Source: Genes (Basel). 2025 Oct 26;16(11):1262. doi: 10.3390/genes16111262 (PMC12651991; doi:10.3390/genes16111262)
Supplement: Supplementary file 1 [file genes-16-01262-s001.zip › Table S2.pdf]

Table S2. Comparison Results of Ty1-*copia* Transposon in NCBI.

***Tamarix chinensis* L.**	**Species**	**Query Cover**	**Similarity**	**E-Value**	**Accession Number**
TC*copia11*	*Coffea eugenioides*	92%	85.49%	3e−75	JF974052.1
TC*copia20*	*Arabis alpina*	99%	89.09%	7e−96	LT669794.1
TC*copia43*	*Lotus japonicus*	100%	92.75%	6e−110	AP022632.1
TC*copia71*	*Coffea kapakata*	100%	86.96%	3e−87	JF974048.1
TC*copia112*	Coffea canephora	92%	85.49%	3e−75	JF974041.1
TC*copia223*	*Coffea canephora*	93%	85.27%	9e−76	JF974041.1
TC*copia225*	*Coffea kapakata*	92%	85.10%	4e−74	JF974048.1
TC*copia229*	*Glycine max*	100%	86.23%	1e−85	AC235914.2
TC*copia281*	*Coffea canephora*	92%	85.49%	3e−75	JF974041.1
TC*copia310*	*Coffea eugenioides*	100%	85.87%	6e−84	JF974034.1
TC*copia343*	*Coffea eugenioides*	100%	85.51%	8e−83	JF974034.1
TC*copia345*	*Coffea canephora*	92%	85.10%	4e−74	JF974041.1
TC*copia425*	*Coffea eugenioides*	100%	85.51%	8e−83	JF974034.1
TC*copia469*	*Coffea canephora*	92%	85.88%	7e−77	JF974041.1
TC*copia491*	*Glycine max*	100%	85.14%	3e−81	AC235857.2
TC*copia509*	*Amaranthus quitensis*	97%	85.35%	3e−81	AF233003.1
TC*copia519*	*Coffea eugenioides*	100%	85.87%	6e−84	JF974034.1
TC*copia549*	*Coffea stenophylla*	100%	85.51%	8e−83	JF974047.1
TC*copia560*	*Coffea canephora*	100%	85.14%	3e−81	JF974037.1
TC*copia566*	*Coffea canephora*	100%	85.25%	1e−80	JF974041.1
TC*copia596*	*Rhizophora apiculata*	99%	91.27%	5e−104	JN715064.1
TC*copia608*	*Coffea canephora*	92%	85.10%	4e−74	JF974041.1
