# Supplementary material for: Characterization of LTR Retrotransposon Reverse Transcriptase in Tamarix chinensis L. and Activity Analysis Under Salt and Alkali Stresses
Source: Genes (Basel). 2025 Oct 26;16(11):1262. doi: 10.3390/genes16111262 (PMC12651991; doi:10.3390/genes16111262)
Supplement: Supplementary file 1 [file genes-16-01262-s001.zip › Table S3.pdf]

Table S3. Comparison Results of Ty3-*gypsy* Transposon in NCBI.

***Tamarix chinensis* L.**	**Species**	**Query Cover**	**Similarity**	**E-Value**	**Accession Number**
TC*gypsy165*	*Cannabis sativa*	99%	90.10%	6e−156	XM_030629513.1
TC*gypsy210*	*Panicum virgatum*	96%	85.61%	4e−127	AC243256.1
TC*gypsy284*	*Solanum pinnatisectum*	100%	91.35%	3e−166	CP047568.1
TC*gypsy333*	*Panicum virgatum*	100%	85.61%	5e−132	AC243256.1
TC*gypsy360*	*Panicum hallii*	99%	92.01%	3e−166	XR_003230036.1
TC*gypsy393*	*Panicum virgatum*	98%	86.65%	7e−136	AC243256.1
TC*gypsy506*	*Panicum virgatum*	98%	85.68%	2e−130	AC243256.1
TC*gypsy597*	*Solanum tuberosum*	100%	92.07%	7e−174	CP046701.1
